# Kids Out; evaluation of a brief multimodal cluster randomized intervention integrated in health education lessons to increase physical activity and reduce sedentary behavior among eighth graders

**DOI:** 10.1186/s12889-019-6737-x

**Published:** 2019-04-17

**Authors:** M. Aittasalo, A-M Jussila, K. Tokola, H. Sievänen, H. Vähä-Ypyä, T. Vasankari

**Affiliations:** UKK Institute for Health Promotion Research, PO Box 30, 33501 Tampere, Finland

**Keywords:** Adolescents, School, RE-AIM, Health education, Lessons, Health promotion, Internet

## Abstract

**Background:**

Most Finnish adolescents are not sufficiently physically active. Health education (HE) provides beneficial starting point for physical activity (PA) promotion in schools. This study evaluates an intervention integrated into three HE lessons to increase PA and reduce sedentary behavior (SB) among eighth graders.

**Methods:**

All public secondary schools in Tampere, Finland participated and were randomized to intervention (INT, *n* = 7) and comparison group (COM, *n* = 7). In INT (690 students, 36 classes) the teachers (*n* = 14) implemented behavioral theory-driven content during three HE lessons. In COM (860 students, 41 classes) the teachers (*n* = 14) carried out standard lessons. The evaluation was based on RE-AIM: **E**ffectiveness was assessed from baseline to 4 weeks (Follow-up 1) and **M**aintenance from 4 weeks to 7 months (Follow-up 2) with change in students’ PA and SB and related psychosocial and parental factors. Methods included questionnaire, accelerometer and activity diary. Linear mixed models with baseline adjustments and random effect correction were used to compare the difference in change between INT and COM. Data on **R**each, **A**doption and **I**mplementation were collected during the process.

**Results:**

Intervention effects were only seen in the self-reported data favoring INT in the weekly number of days with at least 1 h of brisk leisure PA (0.3 [95%CI 0.1 to 0.6]), proportion of students meeting PA recommendations (4.1 [95%CI 2.5 to 5.7]), proportion of students reporting that their family sets limitations for screen time (5.4 [95%CI 3.3 to 7.4]) and in the number of days on which the students intended to do leisure PA in the following week (0.3 [95%CI 0.1 to 0.6]). The effects on PA were still beneficial for INT at Follow-up 2. The intervention reached 96% of the students, was adopted in all 7 schools and was implemented by 13/14 teachers in 35/36 classes.

**Conclusions:**

The intervention was feasible and had small favorable effects on students’ self-reported PA, intention to do PA and family norm in screen time. The effects on PA persisted until Follow-up 2. It is likely that for greater impacts the HE lessons should have been supported with other actions without compromising feasibility.

**Trial registration:**

NCT01633918 (June 27th, 2012).

**Electronic supplementary material:**

The online version of this article (10.1186/s12889-019-6737-x) contains supplementary material, which is available to authorized users.

## Background

Majority of adolescents in Finland and many other European countries are not sufficiently physically active [1, 2]. This is alarming since physical activity (PA) is associated positively with adolescents’ immediate [3, 4] and future health [5–7]. Furthermore, the patterns of PA in adolescence are also likely to transfer to adulthood [8] and in adults, physical inactivity is one of the leading causes of ill-health and death [9]. Actions are therefore needed to promote PA and reduce sedentary behavior (SB) among adolescents.

During the past decade schools have been harnessed to tackle diverse public health issues [10] such as physical inactivity. School reaches majority of children and adolescents regardless of their family background or life circumstances, covers half of their daily waking hours throughout the year and encloses many years [11] during which the lifestyles are still modifiable [12].

Most scientific reviews conclude that school-based interventions may be effective in promoting adolescents’ PA although the effects have generally been small (e.g. [13, 14]). Especially multicomponent whole-school approaches have shown promising results [15, 16] but contradictory findings have also been reported [17]. The effects of information and communication technology in conjunction with other delivery modes seem also encouraging [18].

In Finnish secondary schools (grades 7–9, ages 13–15) health education (HE) provides an exceptionally favorable platform for PA promotion. It is a stand-alone compulsory subject with 3 courses each including 38 lessons [19]. The content is guided by the National Core Curricula, which can be locally adapted [20]. The main themes are growth and development; health in everyday choices; resources and coping skills; and health, society and culture. To qualify, the teachers need to undergo 60 university credits in health sciences. [19]. According to WHO Health Behavior in School-aged Children (HBSC) -survey, the perceptions of Finnish 13–15-year old students about HE lessons are primarily positive: 86% of girls and 79% of boys agree that HE lessons teach them to think about the pros and cons related to various health behaviors [21].

HE is also part of national or state curricula in some other EU countries and for example in the United States and Canada. In these countries, HE is most commonly incorporated into other subjects such as physical education, social studies or specific health programs. [20]. Regardless of its univocal potential no publications on utilizing curriculum-based HE in PA promotion can be found in Finland or internationally.

This paper reports the RE-AIM evaluation [22] of a randomized controlled Kids Out! -intervention, which aimed to promote PA and reduce SB among eighth graders by integrating behavioral theory-driven content into three routinely scheduled HE lessons in secondary schools. The multimodal content included Internet-based self-assessment with feedback views, YouTube-video, refillable student leaflet, refillable classroom poster, classroom peer-discussions and parental leaflet. It was hypothesized that the intervention would impact positively on behavior as well as psychosocial and parental factors related to students’ PA and SB.

## Methods

Full description of the methods is provided in the protocol article [23]. The study plan was approved by the Ethics Committee of the Tampere Region, under the auspices of University of Tampere, Human Sciences (https://www.tuni.fi/en/research/responsible-research/ethical-reviews-in-human-sciences#show-the-request-for-ethical-review--id1889, running number 6/2012). The study has been registered at ClinicalTrials.gov (NCT01633918). The students’ participation in the data collection was based on a written informed consent. A parent or other guardian was to sign student’s consent to make sure that the family was aware of the procedure and student’s decision about data collection. The parents were also informed about the study via electronic communication system between school and home. The CONSORT checklist extended for cluster randomized controlled trials and TIDieR checklist for intervention description and replication are presented in Additional file 1.

### Participants

All 14 public secondary schools in Tampere participated in the study. They were arranged into pairs according to the information (number of students, location [urban/suburban], proportion of students walking or cycling to school) obtained from previous survey to eighth graders [24]. The schools in each pair were then randomized into either intervention (INT, *n* = 7) or comparison group (COM, *n* = 7). Classes or students were not randomized in individual schools to avoid contamination of the groups.

### Intervention

In INT a new content on PA guided by the Health Action Process Approach -model [25] was integrated into three routinely scheduled HE lessons (Lessons #1–3, Table 1). All teachers in INT received one-hour training from the researchers and a Teacher’s Manual to deliver the lessons. The manual included detailed description of the contents and material for each lesson; class-specific student lists for each lesson, where the teachers were asked to keep record of students’ attendance; and space for additional notes or comments.

**Table 1:** The structure and contents of the health education lessons guided by the Health Action Process Approach [25]

Procedure	Contents	Elements of HAPA
ORIENTATION PHASE		
Lesson 1: Orientation	- Teacher presents the intervention and informs about the SoftGIS questionnaire - Students complete the SoftGIS questionnaire via internet (https://maptionnaire.com) - Homework 1 for the next lesson: FeetEnergy-homework leaflet and instructions	- attitudes
MOTIVATIONAL PHASE: INTENTION BUILDING		
Homework 1 - Me & PA	- FeetEnergy-homework leaflet, part 1: Self-assessment of time spent in a) active commuting to school, b) moderate-intensity physical activity (PA) and c) sedentary behavior (SB) and self-conclusion about meeting the recommendations for health.	- attitudes
Lesson 2: Me, peers & PA	- Teacher shows three feedback views based on the school-specific SoftGIS responses and leads discussion on the views: View 1: active commuting to school Map of the city of Tampere with a dot indicating the school ⇒ proportion of students by gender and the average minutes of walking or cycling to school within four distance circles from home (less than 1 km, 1-3 km, 3-5 km, more than 5 km) View 2: leisure time PA The quantity of moderate-intensity LTPA on average and by sex View 3: screen time The proportion of students meeting the recommendation of screen-time (≤ 2 h) by sex	- attitudes, outcome expectancies, pre-action self-efficacy, intention
	- Homework for the next lesson: Link to FeetEnergy-video and instructions	- action planning, action self-efficacy
VOLITIONAL PHASE: ACTION PLANNING		
Homework 2 - Recognizing one’s possibilities	- Watching FeetEnergy-video, which introduces PA recommendations and gives tips for increasing PA and decreasing SB - FeetEnergy-homework leaflet, part 2: Making a list of self-selected ways to increase PA and to reduce SB and choosing at least one way for an immediate action plan	- action planning, action self-efficacy
Lesson 3: Goal setting and action planning	- Watching the FeetEnergy-video in the classroom - Discussing in small groups or pairs about the self-selected ways for immediate action plan. - Making the actions visible by writing them on the FeetEnergy-classroom poster - Homework for the next lesson: Writing follow-up comments about the realization of the actions to the space provided in the poster (brief summary in the beginning of lesson 4)	- action self-efficacy

### Lesson #1

Students completed the internet-based SoftGIS questionnaire (currently Maptionnaire, https://maptionnaire.com) [26, 27] in the computer class. The questionnaire uses Geographic Information System (GIS) to enable the respondents to map their school routes, places of PA, modes of transportation and social activity. It also includes two questions on leisure-time PA and screen time. After completing the SoftGIS questionnaire the students received a refillable FeetEnergy-homework leaflet, which included three sections: 1) self-assessment of PA and SB, 2) health-related information on PA and SB and 3) action plan for increasing PA and reducing SB. The students were to complete the first two sections for the next lesson (Lesson #2) and they were given a parental leaflet with similar contents to deliver home.

### Lesson #2

The lesson started with discussions about the school-specific feedback views accumulated automatically from the students’ responses to SoftGIS questionnaire. The three views gave the students an overview on their active commuting to school, weekly moderate-intensity leisure PA and on meeting screen time recommendations. In the end of the lesson, the students received a link to a YouTube video (https://www.youtube.com/watch?v=Q22XOs1DEtM) providing information and ideas for being more physically active and reducing sitting in everyday life. The students were to watch the video and complete the last section of the FeetEnergy-leaflet (action plan for increasing PA and reducing SB) for Lesson #3.

### Lesson #3

The class watched the FeetEnergy-video together and discussed their action plans in the FeetEnergy leaflet in pairs or small groups. After that the students had an opportunity to write their action plans on the FeetEnergy-poster attached to the classroom wall. The lesson ended with teacher’s brief summary about the action plans and a suggestion to finish the poster for the next lesson by writing comments on how the action plans had been accomplished. In the beginning of Lesson #4 the teachers were encouraged to have a brief discussion with the students about the comments.

### Comparison group (COM)

The teachers were asked to reschedule their standard HE lessons on PA to the same time period as in INT. Otherwise, only data collection was carried out in COM and the teachers received the intervention material after the study. Before the intervention a short electronic questionnaire was addressed to the HE teachers (*n* = 28) in INT and COM to clarify what the standard HE practices on PA were at 8th grades. It included questions about the number of HE lessons on PA during the school year; the type of material used during the HE lessons on PA (a list of 9 materials: text book, Internet, booklets or leaflets, videos, activity trackers etc.); and the topics always raised during the HE lessons on PA (a list of 13 topics: PA recommendations, health benefits of PA, injury risks of PA, harmfulness of sitting/screen time etc.).

The response to the questionnaire was obtained from 14 (100%) teachers in INT and 10 (71%) teachers in COM. The mean number of HE lessons on PA during the whole school year varied from 2 to 11 depending on the school. Five schools (3 in INT and 2 in COM) had eight or more HE lessons on PA. There was no statistically significant difference (*p* = 0.68) in the mean number of HE lessons on PA between INT (6.7, SD 5.1) and COM (6.0, SD 2.6). Neither were there differences in the mean number of PA material used in HE lessons (3.5 in INT and 3.8 in COM, *p* = 0.58) nor topics discussed during the lessons (8.6 in INT and 7.3 in COM, *p* = 0.23) between the groups. Thus, the standard practices at baseline seemed comparable in INT and COM.

### Evaluation

**Fig. 1:**
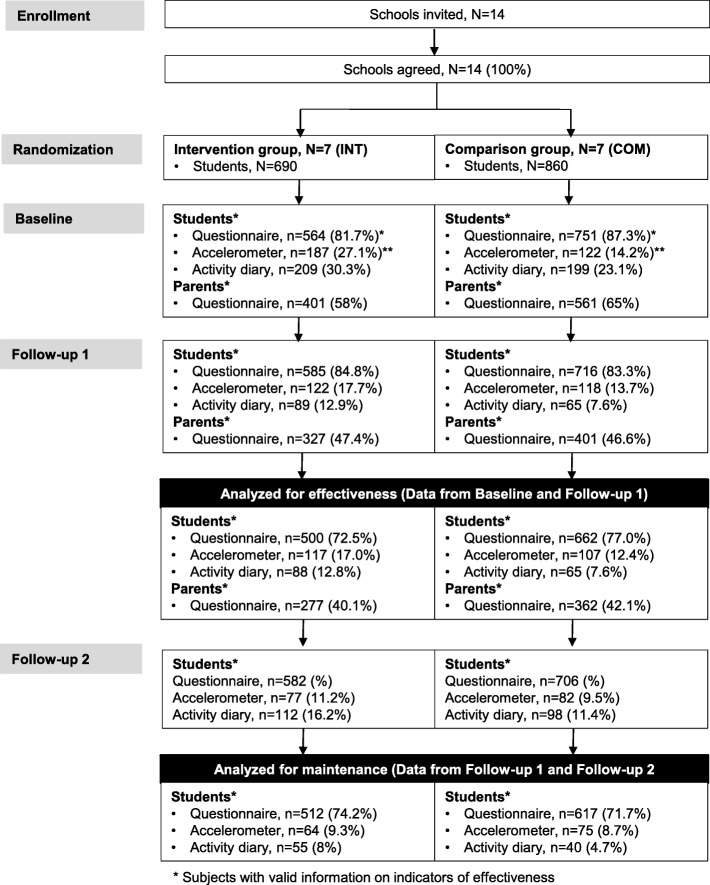
Flow chart of the intervention

The evaluation was based on the RE-AIM framework including five dimensions: **R**each, **E**ffectiveness, **A**doption, **I**mplementation and **M**aintenance [22]. More detailed description of the evaluation questions and indicators as well as timetable of the measurements can be found from the protocol article [23]: Figure 1.

#### Reach

Reach was assessed with the proportion of students participating in at least two of the three intervention lessons (= participants) from the total number of students in INT. The number of students absent from at least two lessons (= non-participants) was obtained from the Teacher’s Manual. To evaluate the representativeness of the participants, their gender, BMI, family circumstances and meeting PA recommendations were compared with the non-participants.

#### Effectiveness — primary indicators

Effectiveness was assessed with change from 1 week before the intervention (Baseline) to 4 weeks after the intervention (Follow-up 1). The primary self-reported indicators of effectiveness were drawn from a questionnaire, which the students completed during one school lesson under the supervision of a teacher: main transportation mode to school, weekly number of days walking or cycling to school, weekly number of days with at least 1 h of brisk leisure PA, participation in organized sports and weekly number of days with more than 2 h of screen time. The indicator-specific questions and their response alternatives are presented in Additional file 2*.* The questions on weekly frequency of walking and cycling to school, brisk leisure PA and screen time were formatted similarly to WHO HSBC –survey showing acceptable reliability in a similar age group of Australian [28] and Finnish adolescents [29].

For objective assessment of effectiveness, an accelerometer (Hookie AM20, Traxmeet Ltd., Espoo, Finland) was offered to all the students at the same measurement points as the questionnaire. The students received written instructions to wear the accelerometer on their right hip during waking hours for 7 days from Monday to Sunday and to remove it only for sauna, shower and water activities. The device has been found as valid as the most commonly used accelerometer (Actigraph GTX3, Actigraph LLC, Pensacola FL, USA) in assessing adolescents’ PA and SB [30]. After both measurement periods the accelerometer-users in both groups received an individual graphical feedback about their daily amount of intensity-specific PA and SB and meeting the PA recommendations.

A 7-day activity diary illustrated in Additional file 3 was used to estimate PA and SB in specific contexts such as ‘walking or cycling to/from school’, ‘PA/SB during school hours’ and ‘participation in organized sports’. Each day at 9 p.m. a text message was sent to the students and/or to one of the parents on to remind them of completing the diary entries from the same day and of using the accelerometer on the next day. The mobile phone numbers were obtained from the informed consents.

At baseline one school in COM with 130 students was not able to arrange the distribution of accelerometers and activity diaries and thus only questionnaire data was collected at all measurement points.

#### Effectiveness — secondary indicators

Psychosocial factors (family norm, short-term behavioral intention, confidence to execute the behavioral intention) related to walking or cycling to school, leisure PA and screen time were assessed to examine the possible changes underpinning behavior change. The indicators were chosen based on previous reviews, which show that self-efficacy, intention to change behavior and family support are among the most powerful correlates [31] and mediators of change [32] behind adolescents’ PA. The questions related to the psychosocial indicators are presented in Additional file 4*.* The questions on short-term behavioral intention and confidence to execute the intention were obtained and translated from Roberts et al. [33], where the corresponding items were named ‘Goal intention’ and ‘Task-efficacy’.

Parental factors were included in the effectiveness evaluation because minimal effort was made via parental leaflet to engage parents to influence their child’s activity patterns. Each student delivered a questionnaire to their parents at Baseline and Follow-up 1 and returned the completed questionnaire back to school in a sealed envelope. The background section included questions on e.g. respondent’s relationship with the student (mother/father/other), education, working status, weight, height, self-rated fitness, activity level at age 14 to 15, transportation mode to work, and weekly PA and daily sitting. The section with effectiveness indicators included questions on knowledge about PA and screen time recommendations; family discussions on leisure PA, school commuting and screen time; and family efforts to influence child’s leisure PA, walking and cycling to school and screen time. The items were chosen after exploring previous studies about the parental correlates of PA in this age group [34, 35] and the questions were developed particularly for this study because no valid measures for evaluating change in the items were found (Additional file 5).

#### Adoption

The adoption rate indicates the extent to which the setting participates in the intervention [22]. This was assessed with the proportion of public schools participating in the study and eventually completing the study. The assessment was based on process evaluation.

#### Implementation

Implementation evaluation comprises various aspects of program delivery such as fidelity, dose delivered, dose received, quality, responsiveness, differentiation and adaptation [36]. The present study focused on fidelity and responsiveness. Safety was also included since it was considered important for further transferability.

Fidelity was assessed with the proportion of teachers delivering the intervention, the proportion of lessons delivered and the proportion of parents recalling receiving PA material from school. Data on the first two were obtained from the Teacher’s Manual. Information on parental recollection was drawn from the Follow-up 1 questionnaire, where the parents were asked whether they recalled receiving any material from school about PA (yes/no) and to name the material.

Responsiveness included teachers’ perceptions about the applicability of the lessons and acceptability of the material (student’s FeetEnergy-leaflet, parents’ FeetEnergy-leaflet, FeetEnergy-poster, FeetEnergy-video). The teachers answered a semi-structured telephone-interview at Follow-up 2 (7 months from baseline) about how well each lesson and material had met their purpose (1 = extremely poorly… 5 = extremely well).

Safety was assessed with the student questionnaire by comparing the change in the proportion of students with PA restrictions between INT and COM from Baseline to Follow-up 1. The question addressed to the students was “Do you have any illness, injury or other restriction, which impairs or prevents you from being physically active?”

#### Maintenance

Two years has been suggested as a minimum length of time for evaluating maintenance [22]. This rule was applied to evaluate the maintenance at organizational level. The indicator was the extent the teachers in INT still used the material developed for the HE lessons 2 years after the intervention. For this purpose, the teachers were e-mailed an electronic questionnaire.

Due to practical and financial reasons the two-year rule could not have been followed in evaluating maintenance at individual level. Instead, the purpose was to see whether the effects on behavior and psychosocial factors possibly discovered at Follow-up 1 (4 weeks from baseline) were maintained until Follow-up 2 (7 months from baseline). This was assessed with between-group difference in change from Follow-up 1 to Follow-up 2 only in variables, which showed statistically significant effects at Follow-up 1. Previous studies show that if the intervention is not effective in short-term, the likelihood for discovering signs of effectiveness in long-term diminishes [37].

### Statistical methods

Power calculations were based on previous survey to eighth graders in Tampere [24]. The calculations applied intra-cluster correlations of 0.005 and 0.01, standard deviation of change of 2.0, significance level of 0.05 and power of 80%. Based on them, 54 to 74 students from each secondary school were needed to discover the between-group difference of 0.5 days in the weekly number of days with more than 2 h of screen time and in the weekly number of days with at least 1 h of brisk leisure PA.

Descriptive data is presented in proportions (%), means and standard deviations (SD). The analysis of **E**ffectiveness (from Baseline to Follow-up 1) and **M**aintenance (from Follow-up 1 to Follow-up 2) included only participants, who had data from both measurement points. Linear mixed models adjusted for age, sex and baseline value and teacher as a random effect were used to compare the difference in change between INT and COM. For dichotomous variables such as meeting PA recommendations (yes/no) difference in change between the groups in %-points with 95% confidence intervals was calculated. However, this procedure allowed no adjustments or random effect correction. Therefore, the statistical levels were re-checked with generalized linear mixed models (GLMM) with same adjustments and random effect correction as used in analyzing non-dichotomous variables. All statistical analyses were conducted using IBM SPSS statistics software (IBM SPSS Statistics for Windows, Version 25.0. Armonk. NY: IBM Corp).

Accelerometer-specific cut-points, which have been previously determined from adolescents’ raw acceleration data by using mean amplitude deviation (MAD), were used to classify the intensity of PA (light, moderate-to-vigorous) and to separate SB (sitting + reclined posture) from PA [30]. Transition from sitting to standing posture (breaks from sitting) was based on the number of SB periods ending with standing up. Standing was detected if the MAD value was greater than 50 mg for the preceding or same epoch when the measured posture changed to standing [38]. Data from at least 4 days with the minimum of 10 h wear-time was considered sufficient to describe students’ PA and SB during regular week. The corresponding criterion for valid diary data was entries from at least three full days. Wear-time was added in the adjustments (age, sex, baseline value) in analyzing accelerometer-based between-group differences in change.

## Results

### Reach

The total number of eighth graders in the 14 public secondary schools was 1550 (Fig. 1). Of them, 690 (44.5%) studied in the 36 classes of INT, where the intervention was implemented as part of the routine school curriculum. Thus, every student in INT was obliged to attend the intervention lessons and eligible to participate.

According to the Teacher’s Manual the students’ participation rates in Lesson #1, Lesson #2 and Lesson #3 were 92.6, 93.5 and 90.4%, respectively. The school-specific rates for each lesson varied from 83.7 to 97.1%, from 90.9 to 97.1% and from 75.8 to 96.3%, respectively. Altogether 30 (4.3%) students missed at least two of the three lessons (non-participants) — their proportion ranged from 1.9 to 8.1% in individual schools. Due to the low number of non-participants statistically significant differences could not be detected between the participants and non-participants in gender, body mass index (BMI) for age (underweight, normal weight, overweight) [39], meeting PA recommendation and family circumstances (living with both parents or in a blended family, with a single parent, with another person). However, the non-participants appeared more likely to be boys (61.9% vs. 51.4%), to live with a single parent (46.6% vs. 28.7%), to meet PA recommendation (20.0% vs. 7.4%) and to be overweight (20.0% vs. 12.3%).

### Effectiveness

Of the 1550 students 1476 (95.2%) responded to the baseline questionnaire. Their distribution by gender, meeting PA recommendation, being overweight and living with a single parent were comparable with population-based data in Tampere and nationally [40]. There were no substantial baseline differences between INT and COM in any of the students’ background variables (Table 2). The parental baseline data seemed also similar in both groups with an exception that in comparison with INT the parents in COM were slightly more often overweight and rated less often their fitness better than peers (Table 3).

**Table 2:** Background characteristics of the students responding to the baseline questionnaire in the intervention (INT) and comparison (COM) group. Means and standard deviations (SD) or numbers (n) and proportions (%)

	INT (*n* = 651)	COM (*n* = 825)
Age (years), mean (SD)	13.9 (0.5)	13.9 (0.5)
Girls, n (%)	314 (48.2)	391 (47.4)
Body mass index^a)^, mean (SD)	19.8 (2.7)	19.8 (2.8)
• > 25 kg/m^2^, n (%)	62 (12.5)	80 (12.5)
Self-rated fitness, n (%)		
• Better than peers	198 (35.6)	242 (32.8)
• Equal to peers	235 (42.3)	322 (43.8)
• Worse than peers	123 (22.1)	172 (23.4)
Distance from home to school, mean (SD)	4.5 (5.5)	4.2 (4.8)
• < 1 km, n (%)	68 (12.5)	116 (15.8)
• 1–3 km	287 (52.7)	328 (44.8)
• > 3 ≤ 5 km	53 (9.7)	93 (12.7)
• > 5 km	137 (25.1)	195 (26.6)
Main residency, n (%)		
• Apartment house	171 (30.5)	235 (31.7)
• Detached house	209 (37.3)	286 (38.5)
• Row house or semi-detached house	153 (27.3)	184 (24.8)
• Combination of two from the residence types above	22 (3.9)	26 (3.5)
• Other	5 (0.9)	11 (1.5)
Family circumstances, n (%)	22 (3.9)	26 (3.5)
• Living with both parents or in a blended family	393 (70.2)	490 (65.9)
• Living with a single parent (permanently or rotating between parents)	163 (29.2)	244 (32.8)
• Living with another person	4 (0.7)	8 (1.1)
Having a car in the family, n (%)	513 (91.6)	675 (91.5)
• Number of cars, mean (SD)	1.5 (0.9)	1.5 (0.8)
Having a moped, scooter or microcar, n (%)	54 (9.7)	67 (9.1)
Having a rideable bicycle, n (%)	510 (91.6)	675 (91.3)

^a)^BMI-for-age [39]

**Table 3:** Background characteristics of the parents responding to the baseline questionnaire in the intervention (INT) and comparison (COM) group. Means and standard deviations (SD) or numbers (n) and proportions (%)

	INT (*n* = 401)	COM (*n* = 561)
Relationship with the student, n (%)		
• Mother	317 (79.1)	436 (77.7)
• Father	77 (19.2)	116 (20.7)
• Other	7 (1.7)	9 (1.6)
Education, n (%)		
• Secondary school	13 (3.3)	17 (3.0)
• High school graduate	15 (3.8)	26 (4.6)
• Vocational school	195 (48.9)	289 (51.7)
• Lower or higher university degree	176 (44.1)	228 (40.7)
Working full or part-time, n (%)	332 (82.2)	481 (85.9)
BMI, mean (SD)	24.8 (4.3)	25.4 (4.3)
• > 25 kg/m^2^, n (%)	145 (37.9)	253 (46.8)
Self-rated fitness		
• Better than peers	194 (48.7)	232 (41.7)
• Equal to peers	149 (37.4)	227 (40.8)
• Worse than peers	54 (13.6)	98 (17.6)
Own activity level at age 14 to 15 on Osgood scale from 0 to100^a)^, mean (SD)		
• Active commuting to school	6.9 (3.0)	6.9 (3.1)
• Exercise as a hobby	6.6 (2.8)	6.5 (3.2)
Walking or cycling as a primary transportation mode to work, n (%)	85 (21.4)	116 (20.8)
Frequency of moderate-to-vigorous leisure PA		
• ≥ 4 times a week	143 (35.7)	195 (34.8)
• 2 to 3 times a week	189 (47.1)	233 (41.5)
• ≤ once a week	69 (17.2)	133 (23.7)
Daily minutes of sitting during a weekday, mean (SD)		
• At work	235 (171)	222 (169)
• At home (TV, videos, computer)	117 (59)	114 (72)

^a)^0 means not at all active and 100 means very much active

Effectiveness analysis included only students and parents, who had data from both baseline and Follow-up 1. Thus, the proportion of students analyzed for the effectiveness was 1162 (75%) in the questionnaire data, 224 (14.5%) in the accelerometer data and 153 (9.9%) in the activity diary data (Fig. 1). The parental data for effectiveness evaluation included 639 (41.2%) parents. In comparison with those not included in the analysis fewer students in the questionnaire analysis were underweight (14% vs. 7%, *p* = 0.014) and more students in the activity diary analysis rotated between parents or lived with a single parent (29% vs. 17%, *p* = 0.026). Moreover, fewer parents in the analysis belonged to the group engaging in moderate-to-vigorous PA once a week or less often (19% vs. 26%, *p* = 0.018).

#### Primary indicators

In the self-reported data, a small intervention effect favoring INT was seen in weekly number of days with at least 1 h of brisk leisure PA (0.3 [95%CI 0.1 to 0.6]) and in the proportion of students meeting PA recommendations (4.1 [95%CI 2.5 to 5.7]) (Table 4). The latter persisted after GLMM. In the accelerometer or activity diary data, no statistically significant differences were found between the groups (Table 5).

**Table 4:** Questionnaire-based primary indicators of effectiveness at baseline (B) and 4 weeks after the intervention (F1) in the intervention (INT) and comparison (COM) group and the differences in change between the groups from B to F1 and 95% confidence intervals (95% CI)

	INT		COM		Between-group difference in change (95% CI)^a)^
	B	F1	B	F1	
n	564	585	751	716	INT = 500; COM = 662
Walking or cycling to school					
• Walking or cycling as a primary transportation mode to school, n (%)	302 (53.5)	273 (46.8)	398 (53.1)	347 (48.6)	−2.8 (−6.2 to 0.6)
• Weekly number of days walking or cycling to school (0–5 days), mean (SD)	3.0 (2.3)	2.8 (2.3)	3.0 (2.4)	2.8 (2.4)	−0.0 (−0.2 to 0.2)
Leisure PA					
• Weekly number of days with at least 1 h of brisk leisure PA (0-7 days), mean (SD)	3.6 (2.0)	3.7 (1.9)	3.5 (1.9)	3.4 (1.9)	0.3 (0.1 to 0.6)^**b)**^
• Meeting PA recommendations (yes), n (%)^c)^	43 (7.7)	53 (9.2)	50 (6.7)	34 (4.8)	4.1 (2.5 to 5.7)^**b)**^
Participation in organized sports (yes), n (%)	204 (36.6)	202 (35.1)	272 (36.9)	274 (38.8)	−1.0 (−2.1 to 0.1)
• Times per week, mean (SD)	2.4 (2.3)	2.4 (2.4)	2.1 (2.1)	2.2 (2.3)	−0.0 (−0.2 to 0.1)
• Weekly duration, mean (SD)	4.5 (5.5)	4.6 (5.5)	3.8 (4.9)	4.1 (6.0)	−0.0 (− 0.4 to 0.4)
Screen time					
• Weekly number of days with > 2 h of screen time (0–7 days), mean (SD)	3.2 (2.2)	3.1 (2.2)	3.4 (2.2)	3.4 (2.1)	−0.1 (− 0.3 to 0.1)

^a)^Linear mixed models adjusted for age, sex and baseline value; teacher as a random effect. Dichotomous variables were analyzed without adjustments and random effect correction but statistical levels were re-checked with generalized linear mixed models (GLMM) with same adjustments and random effect correction as used in non-dichotomous variables

^b)^Remained statistically significant after GLMM (*p* < 0.05)

^c)^At least 1 h of brisk physical activity on 7 days a week

**Table 5:** Diary and accelerometer-based primary indicators of effectiveness at baseline (B) and 4 weeks after the intervention (F1) in the intervention (INT) and comparison (COM) group and the differences in change between the groups from B to F1 with 95% confidence intervals (95% CI)

	INT		COM		Between-group difference in change (95% CI)^a)^
	B	F1	B	F1	
Accelerometer, n	187	122	170	118	INT = 117; COM = 107
Total PA, mean (SD)					
• Minutes per day	267.1 (53.3)	239.3 (49.7)	256.5 (59.8)	239.2 (60.6)	−8.2 (− 19.3 to 2.8)
• Light-intensity minutes per day	180.9 (34.3)	165.7 (30.7)	176.8 (39.8)	168.8 (38.4)	−6.3 (−14.0 to 1.3)
• Moderate-to-vigorous intensity minutes per day	86.7 (32.3)	73.6 (28.0)	79.7 (30.2)	70.4 (31.4)	−1.1 (−6.9 to 4.8)
• Steps per day	10,238 (3098)	9292 (2704)	9919 (2902)	9141 (3200)	50 (− 813 to 713)
• Breaks from sitting^b)^	39.4 (8.4)	37.6 (7.6)	38.0 (9.1)	37.0 (8.8)	−0.1 (−1.7 to 1.4)
• Meeting PA recommendations^c)^, n (%)	25 (13.4)	17 (13.9)	22 (12.9)	17 (14.4)	−0.2 (−4.5 to 4.0)
Activity diary, n	209	89	199	65	INT = 88; COM = 65
Walking or cycling to school, mean (SD)					
• Minutes per day	7.9 (6.2)	7.7 (6.8)	8.5 (6.2)	8.1 (6.4)	0.7 (−1.3 to 2.7)
• Weekly number of days	3.8 (1.9)	3.3 (2.1)	3.8 (1.8)	3.5 (2.0)	−0.1 (−0.5 to 0.4)
Walking or cycling from school, mean (SD)					
• Minutes per day	9.0 (6.5)	8.3 (7.4)	9.8 (7.2)	9.5 (7.2)	0.5 (−1.9 to 2.9)
• Weekly number of days	3.8 (1.7)	3.3 (2.0)	3.8 (1.7)	3.5 (2.0)	−0.1 (−0.7 to 0.6)
PA during school hours, mean (SD)					
• Total minutes per day	39.7 (23.7)	36.0 (22.9)	33.6 (21.7)	28.6 (17.1)	6.4 (−6.1 to 19.0)
• Minutes per day during recesses	5.5 (9.5)	4.3 (10.5)	9.0 (13.4)	7.9 (11.0)	−0.5 (−3.3 to 2.4)
SB during school hours, mean (SD)					
• Total minutes per day	249.2 (69.9)	260.0 (78.4)	251.9 (78.9)	277.0 (72.7)	−12.4 (−44.4 to 19.6)
Participation in organized sports, mean (SD)					
• Weekly minutes	240.7 (263.4)	267.8 (274.8)	221.9 (281.9)	200.8 (278.9)	5.1 (−58.0 to 68.2)

^a)^Linear mixed models adjusted for age, sex and baseline value; teacher as random effect. Dichotomous variables were analyzed without adjustments and random effect correction but statistical levels were re-checked with generalized linear mixed models with same adjustments and random effect correction as used in non-dichotomous variables

^b)^Number of SB periods ending with standing up

^c)^At least 1 hour of moderate-to-vigorous intensity PA on all valid days

#### Secondary indicators

The intervention affected favorably the proportion of students reporting that their family sets limitations or hopes their child to reduce screen time (5.4%-points [95%CI 3.3 to 7.4]) and the number of days on which the students intended to do leisure PA following week (0.3 days [95%CI 0.1 to 0.6]) (Table 6) Also, the change in the proportion of parents knowing the correct answer in screen time recommendations was higher in INT than in COM (4.8 [95%CI 2.3 to 7.2]) but the statistical significance disappeared after adjustments and random effect correction with GLMM.

**Table 6:** Psychosocial and parental indicators of effectiveness at baseline (B) and 4 weeks after the intervention (F1) in the intervention (INT) and comparison (COM) group and the differences in change between the groups from B to F1 with their 95% confidence intervals (95% CI)

	INT		COM		Between-group difference in change (95% CI)^a)^
	B	F1	B	F1	
Psychosocial indicators (Student questionnaire), N	561	585	749	716	INT = 486; COM = 646
Family norm, n (%)					
• Walking or cycling to school (more often or as often as now)	265 (47.9)	327 (56.6)	354 (47.9)	372 (52.4)	3.6 (0.5 to 6.6)^**b)**^
• Leisure PA (more often or as often as now)	449 (80.2)	448 (77.4)	595 (80.2)	540 (76.2)	−0.4 (−2.4 to 1.6)
• Screen time (sets limitations or hopes for reduce)	328 (59.3)	339 (59.2)	448 (60.7)	379 (53.9)	5.4 (3.3 to 7.4)^**c)**^
Short-term behavioral intention, mean (SD)					
• Walking or cycling to school (0–5 days)	3.0 (2.3)	2.7 (2.3)	3.0 (2.4)	2.7 (2.4)	0.0 (−0.2 to 0.3)
• Leisure PA (0–7 days)	3.6 (2.0)	3.7 (1.9)	3.5 (1.9)	3.4 (1.9)	0.3 (0.1 to 0.6)
• Exceeding 2 h of screen time (0–7 days)	2.9 (2.2)	2.8 (2.2)	3.0 (2.2)	3.1 (2.2)	−0.1 (− 0.4 to 0.1)
Confidence to execute the short-term intention, n (%)					
• Walking or cycling to school (totally or quite confident)	455 (82.4)	482 (84.0)	632 (85.3)	595 (84.2)	2.6 (1.3 to 3.9)
• Leisure PA (totally or quite confident)	518 (92.5)	523 (91.4)	701 (94.2)	640 (90.8)	1.5 (−0.3 to 3.4)
• Reducing screen time if wanted (totally or quite confident)	446 (81.4)	482 (84.8)	618 (84.2)	594 (84.8)	1.5 (0.0 to 3.1)
Parental indicators (Parental questionnaire), N	401	327	558	401	INT = 277; COM = 362
Knowledge about PA recommendations, n (%)					
• Correct answer on PA^**d)**^	365 (90.8)	303 (92.7)	508 (89.0)	356 (88.8)	1.6 (0.0 to 3.2)
• Cannot say	31 (7.7)	23 (7.0)	59 (10.6)	41 (10.2)	0.0 (−1.6 to 1.7)
Knowledge about screen time recommendations, n (%)					
• Correct answer^**e)**^	326 (83.4)	258 (82.4)	447 (81.4)	289 (77.7)	4.8 (2.3 to 7.2)^**2)**^
• Cannot say	12 (3.0)	15 (4.6)	25 (4.4)	30 (7.5)	−0.1 (−2.8 to 2.7)
Family discussions on child’s…, n (%)					
• …leisure PA (yes)	283 (70.6)	190 (58.5)	424 (74.3)	217 (55.4)	4.7 (−2.0 to 11.4)
• …school commuting (yes)	114 (28.8)	49 (15.5)	177 (31.3)	63 (16.5)	0.5 (−5.4 to 6.4)
• …on screen time (yes)	271 (67.8)	201 (62.4)	395 (69.1)	244 (61.9)	2.9 (−1.0 to 6.7)
Family efforts to influence on child’s…, n (%)					
• …leisure PA (yes)	206 (51.8)	118 (37.9)	280 (48.9)	159 (41.7)	−6.0 (−11.5 to −0.6)
• …walking and cycling to school (yes)	50 (12.8)	28 (9.3)	71 (12.6)	28 (7.7)	−0.5 (−4.3 to 3.4)
• …screen time (yes)	227 (57.8)	182 (57.2)	348 (61.3)	224 (57.6)	0.8 (−2.1 to 3.6)

^a)^Linear mixed models adjusted for age, sex and baseline value; teacher as random effect. Dichotomous variables were analyzed without adjustments or random effect correction but statistical levels were re-checked with generalized linear mixed models (GLMM) with same adjustments and random effect correction as used in non-dichotomous variables

^b)^Statistical significance (p < 0.05) did not persist after GLMM

^c)^Remained statistically significant after GLMM (*p* < 0.05)

^d)^All answers ≥60 min a day were considered correct

^e)^All answers < 2 h a day were considered correct

### Adoption

All 14 public secondary schools, which were recruited, agreed and completed the study. Thus, the sample represented all the schools initially enrolled to the study.

### Implementation

#### Fidelity

All the teachers in INT returned the Teacher’ Manual after the intervention. According to their notes, the intervention protocol related to the HE lessons on PA was fully followed by all but one teacher, who failed to implement Lesson #3 in one class with 24 students because the study period of the particular class changed before Lesson #3, and the new study period did not include HE anymore. Thus, the lessons were implemented by 13 of the 14 teachers and in 35 of the 36 classes.

At Follow-up 1 altogether 712 (45.9%) parents – 319 (46.2%) in INT and 393 (45.7%) in COM –responded to the question about whether they recalled receiving some material from school on PA. Of them, 102 (14.3%) gave a positive answer with the number being slightly greater in INT (*n* = 59, 18.5%) than in COM (*n* = 43, 10.9%). Furthermore, 12 (20.3%) of the 59 parents in INT with a positive answer were able to name the material as FeetEnergy -leaflet. This means that 12 (1.7%) out of 690 parents in INT recalled receiving FeetEnergy -material from school.

#### Responsiveness

The interview data about the applicability of the lessons and acceptability of the material was obtained from six teachers in INT at Follow-up 2 (7 months from baseline). The average rating (1–5) for Lesson #1, #2 and #3 was respectively 3.2, 2.6 and 2.4. Correspondingly, students’ FeetEnergy-leaflet, parents’ FeetEnergy-leaflet, FeetEnergy-poster and FeetEnergy-video received average ratings of 3.0, 2.4, 3.2 and 2.8.

#### Safety

At baseline 94 (17.2%) students in INT and 149 (20.6%) students in COM reported restrictions, which impaired or prevented them from being physically active. At Follow-up 1 the corresponding figures were 107 (18.7%) and 153 (22.0%). The difference in change between the groups was − 0.8%-points (95%CI − 3.5 to 1.9), which indicates that the intervention did not elevate the risk for PA restrictions from Baseline to Follow-up 1.

### Maintenance

Only four teachers (three in INT and 1 in COM) responded to the electronic questionnaire 2 years after the intervention. Thus, no proper information was obtained about the organizational maintenance of the intervention.

Assessment of individual level maintenance included only students, who had data from both Follow-up 1 and Follow-up 2. The questionnaire data was available from 1129 (72.8%), accelerometer data from 139 (9%) and activity diary data from 95 (6.1%) of students.

At Follow-up 1 favorable behavioral effects were discovered in INT in the weekly number of days with at least 1 h of brisk leisure PA and in the proportion of students meeting PA recommendations. From Follow-up 1 to Follow-up 2 the effects on brisk PA diluted (0.0 [95%CI -1.6 to 2.5]) and on meeting recommendations disappeared (− 2.8 [95%CI -4.4 to − 1.2]).

## Discussion

The intervention aimed at promoting PA and reducing SB among eighth graders in Tampere, Finland by integrating new behavioral-theory driven content on PA into three routinely scheduled HE lessons in seven public secondary schools randomized to INT. The other seven schools randomized to COM carried out standard HE lessons on PA and participated only in data collection. To our knowledge, the study is the first to conduct a comprehensive RE-AIM evaluation on utilizing HE lessons in PA promotion.

### Key findings

The intervention had a statistically significant effect on students’ self-reported weekly number of days with at least 1 h of brisk leisure PA and on the proportion of students meeting PA recommendation. No behavioral effects were discovered based on the accelerometer or activity diary data. In psychosocial indicators, the intervention seemed to influence positively on family norm of setting limitations for screen time and on students’ intention to do leisure PA following week. (**E**ffectiveness). The behavioral effects diluted but were still partly apparent 7 months after the baseline at Follow-up 2. (individual level **M**aintenance). Two years after the intervention no proper data could be obtained from the teachers via electronic survey about the use of intervention material (organizational level **M**aintenance). Nearly all students participated in at least two of the three intervention lessons. (**R**each). Moreover, all the schools, which were approached, participated in and completed the study. (**A**doption). The intervention lessons were almost fully implemented, the lessons and FeetEnergy -material received moderate rating from the teachers and the intervention did not elevate the risk for PA restrictions. However, only a small faction of parents recalled receiving FeetEnergy -material from school. (**I**mplementation). In summary, the intervention was successful in reach, adoption, implementation (except for parental material) and to some extent also in effectiveness and individual-level maintenance.

### Findings in relation to previous studies

#### Reach

In the recent review the median participation rate of PA interventions in youth was 76% [37]. In relation to this the participation rate of 96% in the present study was excellent resulting most likely from the mandatory nature of HE lessons. As previously shown, incorporating activities into the regular school curriculum may lead to lower attrition and better representativeness than delivery with voluntary commitment [37]. Nevertheless, the characteristics of non-participants and participants are seldom compared in school-based studies. Some studies have found differences in e.g. socioeconomic environment, participation in sports clubs or time spent in sedentary activities but in others no differences have been detected. [37]. In the present study the non-participants did not differ from the participants in a statistically significant way. High participation rate in the intervention lessons and similarity of non-participants and participants indicate that integrating PA promotion in HE lessons may be an efficient and non-selective way to reach the target group.

#### Effectiveness

Previous overview by Naylor and McKay [11] on preventing inactivity in schools indicates that classroom-based education alone is not able to increase adolescents’ PA levels but if supplemented with self-assessment and tracking may be effective in reducing screen time. The findings of the present study, which included the supplementary elements mentioned above, did not support this view. Most reviews on school-based PA promotion advocate whole-school approaches employing multilevel strategies [14, 16]. It is thus likely that in order to change PA or SB behavior, the HE lessons of the present intervention should have been supported with environmental and policy strategies, community involvement and more powerful linkage to the family [37]. However, this would have required more staff and time resources, which cannot be easily obtained in real-life contexts such as schools.

Naylor and McKay [11] further state that in some studies classroom-based HE has been shown to affect positively psychosocial variables of PA. The findings of the present study supported this view partially as beneficial effects were seen in the student-reported family norm for screen time and students’ intention to do brisk leisure PA following week. However, the knowledge base on the psychosocial factors of PA in adolescence is still quite thin mainly due to heterogeneity of studies [31, 32, 41]. Moreover, the studies on mediators of change in PA [41] and SB [42] are predominantly cross-sectional preventing from making causal conclusions. It is also possible that the mediators differ between subjectively and objectively measured PA [33].

Regarding parental indicators no studies could be found, which examined the effects of school-based intervention similarly to the present study. There are at least three potential explanations for the ineffectiveness of the intervention to influence on parental knowledge, family discussions and family efforts. The first is implementation, which will be discussed further in the implementation section. The second pertains to measures, which may have been too vague to catch potential small changes. The third lies in the intervention itself: the parental leaflet may have been too minimal even for increasing knowledge and involvement.

#### Adoption

In the present study the adoption rate was 100% since all the schools, which were invited, participated and completed the study. Comparison with earlier studies is difficult since adoption is seldom reported in PA interventions among youth [37]. Nevertheless, the adoption rate of the present study indicates that the school staff was highly interested in PA promotion and despite of extra burden, resilient to finish off the actions, which they had been initially signed in.

#### Implementation

It is suggested that the level of implementation affects the outcomes of health promotion interventions [36, 37]. By contrast, Naylor et al. [43] found in their systematic review on school-based PA interventions that in most studies the level of implementation was not linked to changes in PA or SB. The authors, however, state that the lack of linkage does not necessarily mean that there is no connection. The finding may have also resulted from the heterogeneity of implementation and outcome measures. The present intervention showed high fidelity (except in parental material) but only minor effects. In this respect the findings are in line with the previous review and indicate that other issues than implementation were responsible for the modest outcomes.

Factors influencing (e.g. [44, 45]) and strategies enhancing implementation [46] have recently been examined more intensively. Studies indicate, for example, that contextual appropriateness influences the implementation of school-based PA interventions [43, 44]. In the present study the appropriateness was operationalized as responsiveness and included acceptability of the HE lessons and applicability of the material. Both showed only moderate rating but still, the fidelity of the intervention was high in terms of teachers, lessons and classes. Thus, it seems that once the teachers had committed to the intervention, they delivered it regardless of perceived weaknesses in responsiveness.

The moderate-only responsiveness may partly be explained by comments in the Teacher’s Manual, which revealed that some teachers had technical problems with the computers in Lesson#1 and Lesson #2, some had difficulties in facilitating conversation during the lessons and some experienced frustration in capturing the students’ interest in the subject. However, the comments on a specific lesson or material may have been completely opposite between the teachers and also within a single teacher depending on the class she/he had been teaching. This becomes understandable through a separate study, which videotaped some of the intervention lessons and demonstrated, how the flow of a single lesson was affected by the interaction between individual teacher and the students [47]. The finding also justifies the teacher-based random effect correction in the statistical analysis of effectiveness.

Involving teachers and students in the planning process may have led to better responsiveness. In a previous study participation of the target group has been considered important for the applicability, effectiveness, engagement and sustainability of the school-based health promotion programs [48]. Taking teachers’ views into account has been shown to make the approaches more meaningful and relevant to the teachers enhancing the implementation of the intervention [49]. Moreover, students’ participation in school health promotion has been found to have beneficial effects on students themselves, school as an organization, student interaction and student-adult relationship [50].

The poor parental recollection of receiving intervention material from school was likely to be one important reason for the lack of effectiveness in parental knowledge, family discussions and family efforts: no effects can be expected if only a marginal proportion of parents was exposed to the intervention. Involving parents has proved challenging also in other school-based interventions promoting PA in youth (e.g. [51, 52]) although there are few exceptions (e.g. [53]). With this in mind and given that peers seem to have more power over adolescents’ PA than parents [54, 55] it seems worth weighing how much parental involvement is invested in interventions targeting PA promotion in adolescents.

#### Maintenance

Maintenance is generally the least reported dimension in PA interventions among youth mostly because the studies do not usually aim at institutionalizing the interventions beyond the study period [37]. This was also the case in the present study, which included no active strategies to facilitate the use of intervention material after the study. This may be the main reason for not obtaining proper information from the teachers 2 years after the intervention about the use of intervention material to evaluate the organizational maintenance. Other possible explanations for the poor response rate to the electronic questionnaire may have been busy time schedule, lack of interest or being annoyed with one more inquiry related to the study. The teachers may also have been reluctant to give socially undesirable answers about using the material.

At individual level the effects discovered at Follow-up 1 somewhat diluted by Follow-up 2 but were still partly favorable for INT since no statistically significant between-group difference was discovered in the change of weekly number of days with at least 1 h of brisk leisure PA. This is encouraging especially considering the briefness of the intervention. On the other hand, the behavioral effects at Follow-up 1 were small and seen only in the self-reported data. As they further diminished by Follow-up 2 their clinical meaning can legitimately be challenged.

### Strengths

The main strength of the study was the comprehensive evaluation procedure, which is highly recommended for identifying the elements needed to maximize the intervention effects [37] and to translate the study results into practice [37, 56]. Yet, implementation evaluations are still rare in school-based studies [43]. And even if shown implementable in one context, it is presumable that interventions need adaptations in order to be applicable in different circumstances.

Another important strength of the study was the integration of behavior change theory into the intervention lesson. It helped in designing the intervention procedure and content and made the approach more systematic even though it was concurrently recognized that as shown in adults, behavioral theories do not necessarily improve the likelihood of intervention effects [57]. Previous studies on predictors, mediators and explanatory factors of change indicate that the use of theories in adolescents’ PA promotion is uncommon and the variety of theories applied is narrow [58–60]. No studies utilizing Health Action Process Approach -model were found for comparison. It seems that more information is needed on application of behavioral theories in changing adolescents’ PA.

The study used large selection of indicators in the effectiveness evaluation including psychosocial and parental factors. This extended the evaluation perspective to factors, which may precede behavior change especially in brief interventions. It seems, for instance, that behavioral intention has an important role in contributing change in adolescents’ PA [58]. In this sense the findings of the present study, which showed intervention effect on intention to do PA following week, were encouraging.

The original sample covered all the secondary schools, eighth grades and the whole age-cohort in the city of Tampere. This improves the generalizability of the findings to this particular population especially regarding the questionnaire-based data, which at Follow-up 2 still included more than 70% of the original age-cohort. The generalizability is further strengthened by the high adoption and fidelity rate, both enhancing the transferability of the intervention into practice. It therefore seems that the somewhat disappointing findings on effectiveness did not originate from poor external validity of the intervention but from other factors pointed out in more detail in the limitations section.

### Limitations

The major methodological limitation of the study was the high dropout rate from the accelerometer and activity diary measurements at baseline as well as from both follow-ups. Despite the large overall sample size, the small sample sizes in these measures reduced the potential to detect between-group differences and deteriorated the generalizability of findings. On the other hand, relying solely on questionnaire data in interpreting the findings may not have been adequate due to well-known weaknesses of subjective PA measures and due to selectivity issues, which appeared also in this study, although minimally. There is no solid explanation for students’ low interest in using the objective measures in the present study. One reason may be that wearing a hip-mounted accelerometer is considered uncomfortable or laborious. Another reason may be that a diversity of user-friendly monitoring alternatives, which are based on smart phones, are already available for those adolescents interested in their PA and SB.

Use of non-validated questions in psychosocial and parental indicators made it difficult to evaluate the reliability of findings. It may be that the questions did not actually measure the factors they were designed for and / or were not sensitive enough to assess the changes between two time points. Reliable questions are needed in future studies to overcome these deficiencies.

The intervention was brief in terms of generating behavior change. For better effectiveness, it is likely that additional supportive actions and longer intervention period would have been needed. However, this would have inevitably meant more workload for the teachers, which may have threatened the feasibility of the intervention. Presumably, integrating new elements into existing school-curricula is challenging in all countries. In the present study, knowing the challenges restricted the number of intervention lessons to three, which was considered the maximum for feasible implementation. Including supportive actions may have reduced feasibility and prevented from seeing the effects of the lessons alone. Thus, conducting school-based interventions in natural environments is constant balancing between effectiveness and feasibility.

Assessing the quality of implementation on how the teachers implemented the lessons and what the students learned and experienced during the lessons would have provided more insight and explanations to the minor impacts of the intervention. Qualitative data e.g. via interviews or videotaping would also have shown how the elements of Health Action Process Approach, which was the theoretical background of the intervention, actualized in each lesson. Now the gap between high fidelity and modest effectiveness can only be speculated to relate mostly to the briefness of the intervention and partly to the subjective evaluation methods.

## Conclusions

A brief, multimodal intervention, which aimed at promoting PA and reducing SB during three HE lessons, was feasible and had small favorable effects at Follow-up 1 (4 weeks from baseline) on students’ self-reported PA, intention to do PA following week and family norm in screen time. The effects on PA diluted but still partly existed at Follow-up 2 (7 months from baseline). It is likely that in order for the intervention to be more effective, HE lessons on PA should have been supported with other actions without nevertheless compromising feasibility.

## Additional files

Additional file 1: CONSORT 2010 checklist of information to include when reporting a cluster randomized trial and TIDieR checklist for intervention description and replication (DOCX 45 kb)

Additional file 2: Primary indicators of effectiveness and the corresponding questions and response alternatives in the student questionnaire. (DOCX 71 kb)

Additional file 3: Day-specific page from the activity diary (DOCX 265 kb)

Additional file 4: Secondary indicators of effectiveness and the corresponding questions and response alternatives in the student questionnaire. (DOCX 91 kb)

Additional file 5: Secondary indicators of effectiveness and the corresponding questions and response alternatives in the parental questionnaire. (DOCX 75 kb)

## Abbreviations

BMI: Body Mass Index
COM: comparison group
GLMM: generalized linear mixed models
HE: health education
INT: intervention group
PA: physical activity
SB: sedentary behavior
