# Supplementary material for: Kids Out; evaluation of a brief multimodal cluster randomized intervention integrated in health education lessons to increase physical activity and reduce sedentary behavior among eighth graders
Source: BMC Public Health. 2019 Apr 17;19:415. doi: 10.1186/s12889-019-6737-x (PMC6472104; doi:10.1186/s12889-019-6737-x)
Supplement: Supplementary file 1 — CONSORT 2010 checklist of information to include when reporting a cluster randomized trial and TIDieR checklist for intervention description and replication (DOCX 45 kb) [file 12889_2019_6737_MOESM1_ESM.docx]

Additional file 1. CONSORT 2010 checklist of information to include when reporting a cluster randomized trial and TIDieR checklist for intervention description and replication

| Section/Topic | Item No | Standard Checklist item | Extension for cluster designs | Page No * |
| --- | --- | --- | --- | --- |
| Title and abstract | | | |  |
|  | 1a | Identification as a randomised trial in the title | Identification as a cluster randomised trial in the title | N/A |
|  | 1b | Structured summary of trial design, methods, results, and conclusions (for specific guidance see CONSORT for abstracts)^[[1]](#endnote-1),^^[[2]](#endnote-2)^ | See table 2 | p.2:r:26-52 |
| Introduction | | | |  |
| Background and objectives | 2a | Scientific background and explanation of rationale | Rationale for using a cluster design | p.3-4:r.55- 87 |
|  | 2b | Specific objectives or hypotheses | Whether objectives pertain to the cluster level, the individual participant level or both | p.4:r.89-95 |
| Methods | | | |  |
| Trial design | 3a | Description of trial design (such as parallel, factorial) including allocation ratio | Definition of cluster and description of how the design features apply to the clusters | Ref. 23  (Jussila A-M, Vasankari T, Paronen O, Sievänen H, Tokola K, Vähä-Ypyä H, Broberg A, Aittasalo M. KIDS Out! Protocol of a brief school-based intervention to promote physical activity and to reduce screen time in a sub-cohort of Finnish eighth graders. BMC Public Health 2015;15:634. Doi:10.1186/s12889-015-2007-8.) |
|  | 3b | Important changes to methods after trial commencement (such as eligibility criteria), with reasons |  | N/A |
| Participants | 4a | Eligibility criteria for participants | Eligibility criteria for clusters | Ref. 23  p.4:102-106  p.5:112-117 |
|  | 4b | Settings and locations where the data were collected |  | Ref. 23  p. 5-6;r.121-158 |
| Interventions | 5 | The interventions for each group with sufficient details to allow replication, including how and when they were actually administered | Whether interventions pertain to the cluster level, the individual participant level or both | Ref. 23  p 5.-6:r.130-158 |
| Outcomes | 6a | Completely defined pre-specified primary and secondary outcome measures, including how and when they were assessed | Whether outcome measures pertain to the cluster level, the individual participant level or both | p.7-11:r.182-292 |
|  | 6b | Any changes to trial outcomes after the trial commenced, with reasons |  | N/A |
| Sample size | 7a | How sample size was determined | Method of calculation, number of clusters(s) (and whether equal or unequal cluster sizes are assumed), cluster size, a coefficient of intracluster correlation (ICC or *k*), and an indication of its uncertainty | p.11:r.296-301 |
|  | 7b | When applicable, explanation of any interim analyses and stopping guidelines |  | N/A |
| Randomisation: | | | |  |
| Sequence generation | 8a | Method used to generate the random allocation sequence |  | Ref. 23  p.5:r.112-117 |
|  | 8b | Type of randomisation; details of any restriction (such as blocking and block size) | Details of stratification or matching if used | Ref. 23  p.5:r.112-117 |
| Allocation concealment mechanism | 9 | Mechanism used to implement the random allocation sequence (such as sequentially numbered containers), describing any steps taken to conceal the sequence until interventions were assigned | Specification that allocation was based on clusters rather than individuals and whether allocation concealment (if any) was at the cluster level, the individual participant level or both | Ref. 23  p.5:r.112-117 |
| Implementation | 10 | Who generated the random allocation sequence, who enrolled participants, and who assigned participants to interventions | Replace by 10a, 10b and 10c | Ref. 23  p.4:r.102-106  p.5:r.112-117 |
|  | 10a |  | Who generated the random allocation sequence, who enrolled clusters, and who assigned clusters to interventions |  |
|  | 10b |  | Mechanism by which individual participants were included in clusters for the purposes of the trial (such as complete enumeration, random sampling) |  |
|  | 10c |  | From whom consent was sought (representatives of the cluster, or individual cluster members, or both), and whether consent was sought before or after randomisation |  |
|  |  |  |  |  |
| Blinding | 11a | If done, who was blinded after assignment to interventions (for example, participants, care providers, those assessing outcomes) and how |  | Ref. 23  p.5:r.112-117 |
|  | 11b | If relevant, description of the similarity of interventions |  | N/A |
| Statistical methods | 12a | Statistical methods used to compare groups for primary and secondary outcomes | How clustering was taken into account | p.12:r.303-324 |
|  | 12b | Methods for additional analyses, such as subgroup analyses and adjusted analyses |  | p.12:r.303-324 |
| Results | | | |  |
| Participant flow (a diagram is strongly recommended) | 13a | For each group, the numbers of participants who were randomly assigned, received intended treatment, and were analysed for the primary outcome | For each group, the numbers of clusters that were randomly assigned, received intended treatment, and were analysed for the primary outcome | Figure 1.  p.12-16:r.328-438 |
|  | 13b | For each group, losses and exclusions after randomisation, together with reasons | For each group, losses and exclusions for both clusters and individual cluster members | p.12-16:r.328-438 |
| Recruitment | 14a | Dates defining the periods of recruitment and follow-up |  | Ref. 23 |
|  | 14b | Why the trial ended or was stopped |  | N/A |
| Baseline data | 15 | A table showing baseline demographic and clinical characteristics for each group | Baseline characteristics for the individual and cluster levels as applicable for each group | Table2.  Table 3. |
| Numbers analysed | 16 | For each group, number of participants (denominator) included in each analysis and whether the analysis was by original assigned groups | For each group, number of clusters included in each analysis | Table 4.  Table 5.  Table 6.  p.12-16:r.328-438 |
| Outcomes and estimation | 17a | For each primary and secondary outcome, results for each group, and the estimated effect size and its precision (such as 95% confidence interval) | Results at the individual or cluster level as applicable and a coefficient of intracluster correlation (ICC or k) for each primary outcome | Table 4.  Table 5.  Table 6.  p.12-16:r.328-438 |
|  | 17b | For binary outcomes, presentation of both absolute and relative effect sizes is recommended |  | Table 4.  Table 5.  Table 6.  p.12-16:r.328-438 |
| Ancillary analyses | 18 | Results of any other analyses performed, including subgroup analyses and adjusted analyses, distinguishing pre-specified from exploratory |  | Table 4.  Table 5.  Table 6.  p.12-16:r.328-438 |
| Harms | 19 | All important harms or unintended effects in each group (for specific guidance see CONSORT for harms^[[3]](#endnote-3)^) |  | N/A |
| Discussion | | | |  |
| Limitations | 20 | Trial limitations, addressing sources of potential bias, imprecision, and, if relevant, multiplicity of analyses |  | p.22-23:r.621-656 |
| Generalisability | 21 | Generalisability (external validity, applicability) of the trial findings | Generalisability to clusters and/or individual participants (as relevant) | p.23:r.612-619 |
| Interpretation | 22 | Interpretation consistent with results, balancing benefits and harms, and considering other relevant evidence |  | p.18-23:r.470-610 |
| Other information | | |  |  |
| Registration | 23 | Registration number and name of trial registry |  | ClinicalTrials.gov (NCT01633918) |
| Protocol | 24 | Where the full trial protocol can be accessed, if available |  | Ref. 23  (Jussila A-M, Vasankari T, Paronen O, Sievänen H, Tokola K, Vähä-Ypyä H, Broberg A, Aittasalo M. KIDS Out! Protocol of a brief school-based intervention to promote physical activity and to reduce screen time in a sub-cohort of Finnish eighth graders. BMC Public Health 2015;15:634. Doi:10.1186/s12889-015-2007-8.) |
| Funding | 25 | Sources of funding and other support (such as supply of drugs), role of funders |  | p.26:r.705-708 |

** Note: page numbers optional depending on journal requirements*

**
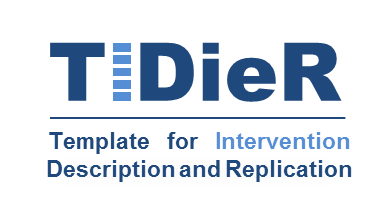
The TIDieR (Template for Intervention Description and Replication) Checklist*:**

Information to include when describing an intervention and the location of the information

| **Item number** | | **Item** | **Where located **** | |
| --- | --- | --- | --- | --- |
|  |  |  | Primary paper  (page or appendix  number) | Other ^†^ (details) |
|  | | **BRIEF NAME** | p.1:r.1-3  p.2:r.26-29  p.4:r.89-95 |  |
| **1.** | | Provide the name or a phrase that describes the intervention. | ____________ | ______________ |
|  | | **WHY** | p.3-4:r 55-87 |  |
| **2.** | | Describe any rationale, theory, or goal of the elements essential to the intervention. | ____________ | _____________ |
|  | | **WHAT** | p.5-6:121-158 | Ref. 23 |
| **3.** | | Materials: Describe any physical or informational materials used in the intervention, including those provided to participants or used in intervention delivery or in training of intervention providers. Provide information on where the materials can be accessed (e.g. online appendix, URL).  URL  SoftGis: [www.softgis.fi/children](http://www.softgis.fi/children)  Feet Energy-material: https://www.tervekoululainen.fi/opetusmateriaalit/tyokaluja-teemoittain/feet-energy-kokonaisuus-liikkeen-lisaamiseen-ja-istumisen-vahentamiseen/  Feet Energy-material: [www.youtube.com/watch?v=Q22XOs1DEtM](http://www.youtube.com/watch?v=Q22XOs1DEtM)  Ref. 23  Jussila A-M, Vasankari T, Paronen O, Sievänen H, Tokola K, Vähä-Ypyä H, Broberg A, Aittasalo M. KIDS Out! Protocol of a brief school-based intervention to promote physical activity and to reduce screen time in a sub-cohort of Finnish eighth graders. BMC Public Health 2015;15:634. Doi:10.1186/s12889-015-2007-8. | ____________ | _____________ |
| **4.** | | Procedures: Describe each of the procedures, activities, and/or processes used in the intervention, including any enabling or support activities.  Ref. 23  Jussila A-M, Vasankari T, Paronen O, Sievänen H, Tokola K, Vähä-Ypyä H, Broberg A, Aittasalo M. KIDS Out! Protocol of a brief school-based intervention to promote physical activity and to reduce screen time in a sub-cohort of Finnish eighth graders. BMC Public Health 2015;15:634. Doi:10.1186/s12889-015-2007-8. | p.5-6:121-158  ____________ | Ref. 23  _____________ |
|  | | **WHO PROVIDED** | p.5:r.121-126 | Ref. 23 |
| **5.** | | For each category of intervention provider (e.g. psychologist, nursing assistant), describe their expertise, background and any specific training given.  Ref. 23  Jussila A-M, Vasankari T, Paronen O, Sievänen H, Tokola K, Vähä-Ypyä H, Broberg A, Aittasalo M. KIDS Out! Protocol of a brief school-based intervention to promote physical activity and to reduce screen time in a sub-cohort of Finnish eighth graders. BMC Public Health 2015;15:634. Doi:10.1186/s12889-015-2007-8. | ____________ | _____________ |
|  | | **HOW** | p.5:r.121-126 | Ref. 23 |
| **6.** | | Describe the modes of delivery (e.g. face-to-face or by some other mechanism, such as internet or telephone) of the intervention and whether it was provided individually or in a group.  Ref. 23  Jussila A-M, Vasankari T, Paronen O, Sievänen H, Tokola K, Vähä-Ypyä H, Broberg A, Aittasalo M. KIDS Out! Protocol of a brief school-based intervention to promote physical activity and to reduce screen time in a sub-cohort of Finnish eighth graders. BMC Public Health 2015;15:634. Doi:10.1186/s12889-015-2007-8. | ____________ | _____________ |
|  | **WHERE** | | p.5-6:r.112-158 | Ref. 23 |
| **7.** | Describe the type(s) of location(s) where the intervention occurred, including any necessary infrastructure or relevant features. | | _____________ | _____________ |
|  | Ref. 23  Jussila A-M, Vasankari T, Paronen O, Sievänen H, Tokola K, Vähä-Ypyä H, Broberg A, Aittasalo M. KIDS Out! Protocol of a brief school-   based intervention to promote physical activity and to reduce screen time in a sub-cohort of Finnish eighth graders. BMC Public Health   2015;15:634. Doi:10.1186/s12889-015-2007-8.  **WHEN and HOW MUCH** | | p.5-6:r.121-158 | Ref. 23 |
| **8.** | Describe the number of times the intervention was delivered and over what period of time including the number of sessions, their schedule, and their duration, intensity or dose.  Ref. 23  Jussila A-M, Vasankari T, Paronen O, Sievänen H, Tokola K, Vähä-Ypyä H, Broberg A, Aittasalo M. KIDS Out! Protocol of a brief school-   based intervention to promote physical activity and to reduce screen time in a sub-cohort of Finnish eighth graders. BMC Public Health   2015;15:634. Doi:10.1186/s12889-015-2007-8. | | _____________ | _____________ |
|  | **TAILORING** | | p.5-6:r.130-158 | Ref. 23 |
| **9.** | If the intervention was planned to be personalised, titrated or adapted, then describe what, why, when, and how. | | _____________ | _____________ |
|  | Ref. 23  Jussila A-M, Vasankari T, Paronen O, Sievänen H, Tokola K, Vähä-Ypyä H, Broberg A, Aittasalo M. KIDS Out! Protocol of a brief school-   based intervention to promote physical activity and to reduce screen time in a sub-cohort of Finnish eighth graders. BMC Public Health   2015;15:634. Doi:10.1186/s12889-015-2007-8.  **MODIFICATIONS** | | N/A (no modifications) |  |
| **10.^ǂ^** | If the intervention was modified during the course of the study, describe the changes (what, why, when, and how). | | _____________ | _____________ |

|  | **HOW WELL** | p.7:r.182-183  p.10:r:258-267 |  |
| --- | --- | --- | --- |
| **11.** | Planned: If intervention adherence or fidelity was assessed, describe how and by whom, and if any strategies were used to maintain or improve fidelity, describe them. | _____________ | _____________ |
| **12.^ǂ^** | Actual: If intervention adherence or fidelity was assessed, describe the extent to which the intervention was delivered as planned. | p.15:r.397-403  _____________ | _____________ |

** **Authors** - use N/A if an item is not applicable for the intervention being described. **Reviewers** – use ‘?’ if information about the element is not reported/not sufficiently reported.

† If the information is not provided in the primary paper, give details of where this information is available. This may include locations such as a published protocol or other published papers (provide citation details) or a website (provide the URL).

ǂ If completing the TIDieR checklist for a protocol, these items are not relevant to the protocol and cannot be described until the study is complete.

* We strongly recommend using this checklist in conjunction with the TIDieR guide (see *BMJ* 2014;348:g1687) which contains an explanation and elaboration for each item.

* The focus of TIDieR is on reporting details of the intervention elements (and where relevant, comparison elements) of a study. Other elements and methodological features of studies are covered by other reporting statements and checklists and have not been duplicated as part of the TIDieR checklist. When a **randomised trial** is being reported, the TIDieR checklist should be used in conjunction with the CONSORT statement (see [www.consort-statement.org](http://www.consort-statement.org)) as an extension of **Item 5 of the CONSORT 2010 Statement.** When a **clinical trial** **protocol** is being reported, the TIDieR checklist should be used in conjunction with the SPIRIT statement as an extension of **Item 11 of the SPIRIT 2013 Statement** (see [www.spirit-statement.org](http://www.spirit-statement.org)). For alternate study designs, TIDieR can be used in conjunction with the appropriate checklist for that study design (see [www.equator-network.org](http://www.equator-network.org)).

1. [↑](#endnote-ref-1)
2. [↑](#endnote-ref-2)
3. [↑](#endnote-ref-3)
