# Supplementary material for: Kids Out; evaluation of a brief multimodal cluster randomized intervention integrated in health education lessons to increase physical activity and reduce sedentary behavior among eighth graders
Source: BMC Public Health. 2019 Apr 17;19:415. doi: 10.1186/s12889-019-6737-x (PMC6472104; doi:10.1186/s12889-019-6737-x)
Supplement: Supplementary file 2 — Primary indicators of effectiveness and the corresponding questions and response alternatives in the student questionnaire. (DOCX 71 kb) [file 12889_2019_6737_MOESM2_ESM.docx]

Additional file 2. Primary indicators of effectiveness and the corresponding questions and response alternatives in the **student** questionnaire

| **Indicator** | **Question and its response alternatives** |
| --- | --- |
| Primary transportation mode to school | What is currently your primary transportation mode to school? Give your respond based on your main residency and choose only one alternative. |
|  | - walking; cycling; by bus; by moped or microcar; by car; other mode, what? |
| Weekly number of days walking or cycling to school | On how many days per week you walk or cycle to one or both ways to school? |
|  | - 0; 1; 2; 3; 4; 5 |
| Weekly number of days with at least 1 hour of brisk leisure PA | On how many days per week do you do physical activity at least for one hour so that you sweat and get slightly out of breath? |
|  | - 0; 1; 2; 3; 4; 5; 6; 7 |
| Weekly number of times participating in organized sports  Weekly duration of participating in organized sports | Do you participate in regular organized sports once a week or more frequently outside school hours? |
|  | - Yes, ______ times per week altogether ______ hours a week; No, I do not |
| Weekly number of days with > 2 hours of screen time | On how many days per week does your screen time exceed two hours? |
|  | - 0; 1; 2; 3; 4; 5; 6; 7 |
