# Supplementary figures and images for: Kids Out; evaluation of a brief multimodal cluster randomized intervention integrated in health education lessons to increase physical activity and reduce sedentary behavior among eighth graders
Source: BMC Public Health. 2019 Apr 17;19:415. doi: 10.1186/s12889-019-6737-x (PMC6472104; doi:10.1186/s12889-019-6737-x)

Additional file 3. Day-specific page from the 7-day activity diary
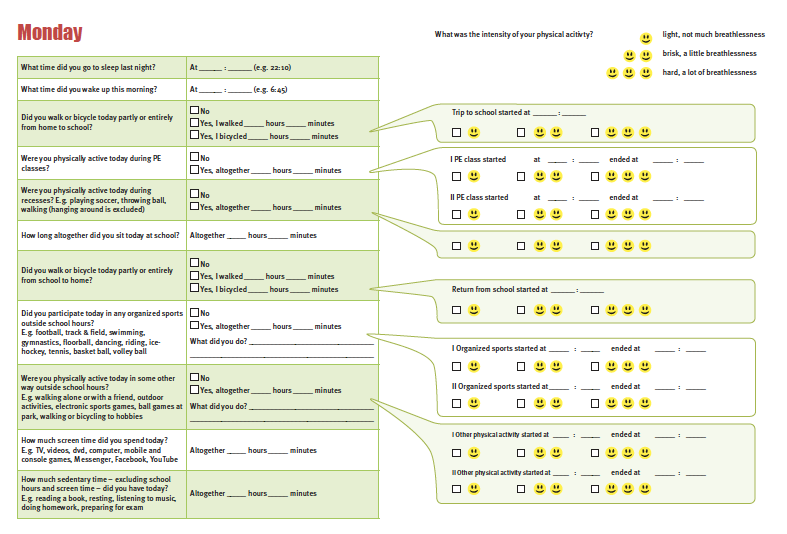

Supplement: Supplementary file 3 — Day-specific page from the activity diary (DOCX 265 kb) [file 12889_2019_6737_MOESM3_ESM.docx]
