# Supplementary material for: Kids Out; evaluation of a brief multimodal cluster randomized intervention integrated in health education lessons to increase physical activity and reduce sedentary behavior among eighth graders
Source: BMC Public Health. 2019 Apr 17;19:415. doi: 10.1186/s12889-019-6737-x (PMC6472104; doi:10.1186/s12889-019-6737-x)
Supplement: Supplementary file 4 — Secondary indicators of effectiveness and the corresponding questions and response alternatives in the student questionnaire. (DOCX 91 kb) [file 12889_2019_6737_MOESM4_ESM.docx]

Additional file 4. Secondary indicators of effectiveness and the corresponding questions and response alternatives in the **student** questionnaire.

| **Indicator** | **Question and its response alternatives** |
| --- | --- |
| **Family norm** |  |
| Walking or cycling to school | My family wants me to walk or cycle to school… |
|  | - more often; as often as now; less often than now; my family does not take a stand |
| Leisure PA | My family wants me to be physically active during leisure… |
|  | - more often; as often as now; less often than now; my family does not take a stand |
| Screen time | My family takes stand on my screen time |
|  | - Yes, my family sets limitations to screen time; Yes, my family hopes me to reduce screen time; No, my family does not take stand. |
| **Short-term behavioral intention** |  |
| Walking or cycling to school | Next week on how many days do you intend to walk or cycle one or both ways to school? |
|  | - 0; 1; 2; 3; 4; 5 |
| Leisure PA | Next week on how many days do you intend to do physical activity at least one hour so that your heartbeat rises? |
|  | - 0; 1; 2; 3; 4; 5; 6; 7 |
| Screen time | Next week on how many days do you intend to exceed two hours of screen time? |
|  | - 0; 1; 2; 3; 4; 5; 6; 7 |
| **Confidence to execute short-term intention** |  |
| Walking or cycling to school | How confident are you that next week you are able to walk or cycle to school on as many days as you intended? |
|  | - not at all confident; quite confident; totally confident |
| Leisure PA | How confident are you that next week you are able to do brisk physical activity on as many days as you intended? |
|  | - not at all confident; quite confident; totally confident |
| Screen time | If you wanted to reduce screen time, how confident are you that you would be able to do that next week? |
|  | - not at all confident; quite confident; totally confident |
