# Supplementary material for: Kids Out; evaluation of a brief multimodal cluster randomized intervention integrated in health education lessons to increase physical activity and reduce sedentary behavior among eighth graders
Source: BMC Public Health. 2019 Apr 17;19:415. doi: 10.1186/s12889-019-6737-x (PMC6472104; doi:10.1186/s12889-019-6737-x)
Supplement: Supplementary file 5 — Secondary indicators of effectiveness and the corresponding questions and response alternatives in the parental questionnaire. (DOCX 75 kb) [file 12889_2019_6737_MOESM5_ESM.docx]

Additional file 5. Secondary indicators of effectiveness and the corresponding questions and response alternatives in the **parental** questionnaire.

| **Indicator** | **Question and its response alternatives** |
| --- | --- |
| Knowledge about PA recommendations | How much do you think that an eight-grader should be physically active on a daily basis?   - Alternatives: _______ hours _______ minutes; I cannot say. |
| Knowledge about screen time recommendations | In your opinion, what is the maximum daily amount of screen time (TV, dvd, computer, game consoles) that an eighth grader can have outside school hours?   - Alternatives: _______ hours _______ minutes; I cannot say. |
|  |  |
| Family discussions on child’s   - leisure PA - school commuting - screen time | During the past month has there been discussion in your family about your child’s leisure PA, school commuting or screen time?   - Alternatives separately for each activity mode: No/Yes |
|  |  |
| Family efforts to influence on child’s   - leisure PA - walking and cycling to school - screen time | During the past month have you made efforts in your family to increase your child’s physical activity or to reduce her/his screen time?   - Alternative No/Yes for each statement: Efforts have been made to get her/him to increase leisure PA; Efforts have been made to get her/him to increase walking or cycling to school; Efforts have been made to get her/him to reduce screen time |
